# Severe Clinical Presentation of Visceral Leishmaniasis in Naturally Infected Dogs with Disruption of the Splenic White Pulp

**DOI:** 10.1371/journal.pone.0087742

**Published:** 2014-02-03

**Authors:** Isadora S. Lima, Joselli S. Silva, Valter A. Almeida, Floriano G. Leal. Junior, Patrício AN. Souza, Daniela F. Larangeira, José P. Moura-Neto, Deborah BM. Fraga, Luiz A. R. de Freitas, Washington L.C. dos-Santos

**Affiliations:** 1 Fundação Oswaldo Cruz, Centro de Pesquisas Gonçalo Moniz, Salvador, Bahia, Brazil; 2 Centro de Referência em Doenças Endêmicas Pirajá da Silva (PIEJ), Jequié, Bahia, Brazil; 3 Universidade Federal da Bahia, Escola de Medicina Veterinária, Salvador, Bahia, Brazil; 4 Universidade Federal da Amazônia, Faculdade de Ciências Farmacêuticas, Manaus, Amazonas, Brazil; Royal Tropical Institute, Netherlands

## Abstract

In this work, we investigated the association between the disruption of splenic lymphoid tissue and the severity of visceral leishmaniasis in dogs. Clinical and laboratory data from 206 dogs were reviewed. Spleen sections collected during the euthanasia of these animals were analyzed, and the splenic lymphoid tissue samples were classified as well organized (spleen type 1), slightly disorganized (spleen type 2), or moderately to extensively disorganized (spleen type 3). Of 199 dogs with evidence of *Leishmania* infection, 54 (27%) had spleen type 1, 99 (50%) had spleen type 2, and 46 (23%) had spleen type 3. The number of clinical signs associated with visceral leishmaniasis was significantly higher in the animals with evidence of *Leishmania* infection and spleen type 2 or 3 than in the animals with spleen type 1. Alopecia, anemia, dehydration, dermatitis, lymphadenopathy, and onychogryphosis were all more frequent among animals with evidence of *Leishmania* infection and spleen type 3 than among the dogs with evidence of *Leishmania* infection and spleen type 1. The association between the severity of canine visceral leishmaniasis and the disorganization of the splenic lymphoid tissue was even more evident in the group of animals with positive spleen culture. Conjunctivitis and ulceration were also more common in the animals with spleen type 3 than in the animals with spleen type 1. The serum levels (median, interquartile range) of albumin (1.8, 1.4–2.3 g/dL) and creatinine (0.7, 0.4–0.8 mg/dL) were significantly lower and the serum levels of aspartate aminotransferase were significantly higher (57, 39–95 U) in animals with spleen type 3 than in animals with spleen type 1 (2.8, 2.4–3.4 g/dL; 0.9, 0.7–1.2 mg/dL and 23, 20–32 U, respectively). Our data confirm the hypothesis that disruption of the splenic lymphoid tissue is associated with a more severe clinical presentation of canine visceral leishmaniasis.

## Introduction

The spleen is the largest secondary lymphoid organ in humans and dogs. The histological structure of the spleen is characterized by a lymphoid tissue known as white pulp, which is immersed in an entangled network of sinusoids, capillaries, and leukocyte-reticular cell cords known as red pulp. The white pulp is organized into 3 different regions: (1) a periarteriolar lymphoid sheath (PALS) around arterioles, (2) discrete nodular lymphoid follicles, and (3) a surrounding and less tightly packed marginal zone. T lymphocytes are the main constituents of the PALS, and B lymphocytes predominate in the other lymphoid areas [1]. Additionally, a complex system of interacting cells comprising different types of macrophages, dendritic cells, and reticular cells is present in these white pulp compartments. This organization of the white pulp permits the transit and differentiation of lymphocytes involved in the response to circulating pathogens and antigens [1], [2]. Hence, at any given time, a histological study of the spleen of normal humans and of normal dogs reveals many secondary lymphoid follicles, which are composed of germinal centers formed predominantly by blastic B lymphocytes organized around a limited number of antigens immobilized in projections of follicular dendritic cells [1], [2], [3]. A rim of inactive B cells that are non-reactive to these antigens remains around these germinal centers. The organization of the white pulp of the spleen also enables the proper positioning of memory cells to respond promptly to T-dependent and T-independent antigens, such as encapsulated bacteria [1], [4]. In both humans and dogs, the absence of the spleen is associated with an increased risk of localized and disseminated infection, including overwhelming bacterial, yeast, or virally induced sepsis [5], [6], [7].

Visceral leishmaniasis and other systemic infections (e.g., viral infections) alter the histological structure of the spleen, leading to disruption of the white pulp microenvironment [8], [9], [10], [11]. Many follicles become atrophic or have low cell density [9]. The usual boundaries between the white pulp compartments and between the white and red pulps are effaced [12]. Previous work by our group and others has shown that disruption of the white pulp structure is more frequent in dogs with laboratory signs of susceptibility to visceral leishmaniasis than in animals in which these susceptibility markers are absent. These signs include a positive spleen culture and a negative leishmanin skin test (LST) against the parasite [12]. We have shown that disruption of the splenic architecture mainly affects the cell populations present in the follicle and in the marginal zone, with a decrease in the number of B cells and follicular dendritic cells in association with a decrease in CXCL13 expression [13]. CXCL13 is a chemokine involved in the maintenance of the lymphoid follicle structure [14], [15], [16]. Recently, Lima and colleagues (2012) reported an association between white pulp disorganization and T-cell apoptosis in the spleens of dogs with visceral leishmaniasis [17]. In that study, dogs with visceral leishmaniasis and a disruption of the splenic tissue had an increased frequency of emaciation and the clinical signs of visceral leishmaniasis. However, the small number of animals used in these studies did not permit the examination of the relationship between splenic disruption and disease severity. In the present study, data from a large number of dogs from an area of endemic visceral leishmaniasis were evaluated to examine the relationship between the histological disorganization of the splenic white pulp and the severity of visceral leishmaniasis. The data presented herein are relevant for understanding the potential mechanisms involved in the genesis of severe forms of visceral leishmaniasis. Furthermore, studies have shown that spleen cellularity can be studied safely by fine needle aspiration [18], [19]. Therefore, the data presented in this work support the possibility of defining markers based on spleen cytology for assessing the severity of visceral leishmaniasis in dogs. Such markers would be useful as complementary tests in trials of therapeutic and immunopreventative reagents and in the clinical follow-up of animals under treatment.

## Materials and Methods

### Ethical Statement

This study was performed in strict accordance with the recommendations of the Brazilian Federal Law on Animal Experimentation (Law 11794) (http://www.planalto.gov.br/ccivil_03/_ato2007-2010/2008/lei/l11794.htm) and with the manual for the surveillance and control of visceral leishmaniasis [20]. The protocol was approved by the Ethics Committee for the Use of Animals in Research (CPqGM-FIOCRUZ, Ceua, license number: 040/2005).

### Animals

The samples and clinical and laboratory data used in this study were obtained from 207 stray dogs of different breeds and different estimated ages that were collected from the streets of Jequié (Bahia State, Brazil, an area of endemic visceral leishmaniasis) between 2004 and 2010. This study was performed in collaboration with the Endemic Diseases Surveillance Program of the State Health Service as part of a program for the surveillance and control of visceral leishmaniasis. All the animals were examined clinically by at least 2 veterinarians using a clinical chart. The presence of anti-*Leishmania* antibodies in the serum was determined by ELISA, and the cellular immune response against *L. infantum* antigens was detected by the LST. Dogs with a positive result by ELISA and those who were not claimed by owners were kept in the kennel for 48 hours with free access to food and water. The dogs were then sedated with acepromazine (0.1 mg/kg iv, Acepram 1%, Vetnil, Brazil) and sodium thiopental (15 mg/kg iv, Thiopentax 1 g, Cristália, Brazil) and euthanized using a saturated solution of potassium chloride (2 mL/kg, iv). Immediately following euthanasia, spleen aspirates were collected for culture, and spleen fragments were collected and frozen in liquid nitrogen for molecular biology studies or fixed in formalin and embedded in paraffin for morphological studies. The technical details of the anti-*Leishmania* ELISA, the LST, and the splenic culture for *Leishmania* isolation have been reported elsewhere [21].

### Clinical Data

All animals were subjected to a clinical exam with an emphasis on parameters considered to be indicative of canine visceral leishmaniasis, defined as follows:

**Alopecia.** Hairless regions at different areas of the skin.

**Anemia.** Pale (light pink) eyes and mouth mucous membranes.

**Conjunctivitis.** Inflammation of the conjunctiva indicated by redness or pus.

**Dehydration.** A poor skin turgor test and/or a delayed capillary refill time.

**Dermatitis.** Dry, exfoliative inflammation of the skin.

**Emaciation.** Skinny, animals with ribs and pelvis evident; cachectic, animals with ribs and pelvis evident and muscle atrophy in the temporal and scapular regions.

**Erosion.** Presence of shallow, moist, or crusted lesions caused by a loss of the epidermis.

**Lymphadenopathy.** Lymph nodes larger than expected for the size of the animal, as follows: dogs weighing less than 5 kg with lymph nodes larger than 2 cm (large axis), dogs weighing 5–10 kg with lymph nodes larger than 3.5 cm, and dogs weighing more than 10 kg with lymph nodes larger than 4 cm.

**Onychogryphosis.** Nails of >1 cm on all extremities.

**Pustules.** Small (2–5 mm) skin vesicles containing pus.

**Splenomegaly.** Animals with a palpable spleen beyond the last rib.

**Ulcerations.** Skin lesions with loss of the epithelial layer, with a granular surface and raised edges.

The animals were grouped into the following categories according to the reported clinical signs suggestive of visceral leishmaniasis: asymptomatic (with no clinical signs) and symptomatic (with any of the clinical signs described above).

### Spleen Samples and Histological Analysis

Spleen tissue slices transverse in orientation to the larger axis of the organ with a thickness of 3 to 4 mm were collected. The tissue slices were fixed in formalin and embedded in paraffin. Hematoxylin- and eosin-stained splenic tissue sections with a thickness of 4 to 5 µm were examined by optical microscopy. The samples were examined by 2 pathologists (LARF and WLCS) and classified into 3 categories (spleen types 1, 2, and 3) according to the degree of structural organization of the splenic white pulp using the criteria previously described by Santana and colleagues (2008) [12]. Briefly, spleen type 1 (well organized) has a distinct periarteriolar lymphocyte sheath, a germinal center, a mantle zone, and a marginal zone; spleen type 2 (slightly disorganized) has either hyperplastic or hypoplastic changes leading to a loss in the definition of the boundaries between regions of the white pulp; spleen type 3 (moderately to extensively disorganized) has white pulp evident but with poorly individualized or indistinct regions or in which the follicular structure was barely distinct from the red pulp and T-cell areas. The last category is frequently associated with lymphoid atrophy.

### Biochemistry and Hematology

Blood samples for hematological and serum biochemical analyses were collected from the cephalic vein of the dogs under manual restraint. The samples were preserved in EDTA-2Na tubes (Greiner Bio-one, Kremsmünster, Austria) and in blood collection tubes (BD Vacutainer®; Becton, Dickinson and Co., BR) and were examined the same day. Total red blood cell (RBC) and white blood cell (WBC) counts were obtained using an automated cell counter (Pentra 80 counter, ABX Diagnostics, Montpellier, France). Microhematocrit tubes containing the samples were centrifuged at 12,000 rpm for 5 min, and the hematocrit was estimated. Differential blood cell counts were also performed. The serum collected by centrifugation in the Vacutainer® tubes was used for the following biochemical tests, using an enzymatic colorimetric method with an A15 auto-analyzer (BioSystems, Barcelona, Spain): total protein (TP), albumin, aspartate aminotransferase (AST), alanine aminotransferase (ALT), total bilirubin, alkaline phosphatase, burea, and creatinine.

### Real-time PCR for the Detection of Leishmania DNA

To detect parasite DNA in frozen spleen samples, a quantitative real-time PCR technique was used. DNA was extracted using a DNeasy® Blood and Tissue Kit (Qiagen, Hilden, Germany) according to the manufacturer’s protocols. A total of 10 mg of spleen tissue was processed following the Qiagen animal tissue protocol. Once extracted, the quality and concentration of each DNA sample was determined using a digital spectrophotometer (NanoDrop® ND-1000, Thermo Scientific, Wilmington, USA). The DNA samples were then adjusted to a concentration of 30 ng/µL, aliquoted, and stored at −20°C until use. Real-time PCR assays were performed using a previously described amplification procedure [22]. The PCR technique targeted a conserved region of *L. infantum* kDNA to obtain a 120-bp amplicon. The reactions were performed in a final volume of 25 µL containing 5 µL of the DNA sample diluted to 30 ng/µL in deionized water and 20 µL of PCR mixture. The PCR mixture consisted of 12.5 µL of Universal Mastermix (Perkin-Elmer Applied Biosystems, Carlsbad, CA, USA), 900 nM each of forward primer 5′-AACTTTTCTGGTCCTCCGGGTAG-3′ (LEISH-1) and reverse primer 5′-ACCCCCAGTTTCCCGCC-3′ (LEISH-2), and a fluorogenic probe (5′-AAAAATGGGTGCAGAAAT-3′), which was synthesized using a FAM reporter molecule attached to the 5′ end and a MGB-NFQ quencher linked to the 3′-end (Perkin-Elmer Applied Biosystems), at a final concentration of 200 nM. A standard curve was generated using serial dilutions of *L. infantum* DNA from 10^6^ to 10^−1^ parasites/mL, and each dilution was performed in triplicate. The amplifications were performed in triplicate for each sample and for the negative control using an ABI Prism 5900 sequence detection system (Perkin-Elmer Applied Biosystems, Carlsbad, CA, USA). A canine housekeeping gene (18S rRNA) was amplified to normalize the concentration of the input sample DNA. The parasite load was expressed as the number of parasites normalized to the established reference amplification value for the 18S rRNA housekeeping gene in 100 mg of host tissue.

### Expression and Significance of the Results

The numerical data shown in the text, tables, and graphs represent absolute values, means, or proportions as indicated. For continuous variables, the significance of the differences between groups were tested using a Mann-Whitney test when there were 2 groups and the Kruskal-Wallis test when more than 2 groups were involved. When the test was significant, the difference between 2 groups was identified using Dunn’s Multiple Comparison test. For comparisons involving proportions, the Chi-square test, the Fisher’s exact probability test, or the Chi-square test for trends were used as recommended [23]. The associations between clinical signs and spleen disorganization were further tested by calculating the odds ratios (OR) using the cross-product ratios of 2×2 tables with 95% confidence intervals [23]. The critical level of significance was established at P<0.05.

## Results

### General Characteristics of the Animals

Of the 207 animals, 1 was excluded because no spleen sample was available for histological study. The main characteristics of the remaining 206 animals are presented in Table 1. At least 1 positive test (PCR, LST, ELISA, or a culture of the spleen aspirate) for *Leishmania* infection was observed in 199/203 (98%) of the animals for which the data was available (Table 2). The clinical signs typically associated with canine visceral leishmaniasis were present in 184 (89%) animals. Among the 206 dogs, 57 (28%) had spleen type 1, 101 (49%) had spleen type 2), and 48 (23%) had spleen type 3 (Table 1).

**Table 1:** General characteristics of stray dogs collected between 2004 and 2010 from the streets of Jequié (Bahia State, Brazil), an area of endemic visceral leishmaniasis.

PARAMETER	FREQUENCY	(%)
N	206	(100)
Gender:		
Male	116/206	(56)
Female	90/206	(44)
Size:		
Small	45/204	(22)
Medium	122/204	(60)
Large	37/204	(18)
Estimated age (years):		
0–2	28/154	(18)
>2–5	102/154	(66)
>6	24/154	(16)
Clinical category:		
Asymptomatic	22/206	(11)
Symptomatic	184/206	(89)
Splenic white pulp organization:		
Type 1	57/206	(28)
Type 2	101/206	(49)
Type 3	48/206	(23)

**Table 2:** Results of laboratory tests for *Leishmania* infection in dogs collected from the streets of an endemic area of visceral leishmaniasis in Brazil.

LABORATORY	TEST AND RESULTS				NUMBER (%)	
CATEGORY	CULTURE^1^	ELISA	LST^2^	PCR^3^		
*Positive:*					*196*	*(95.1)*
	P	P	P	P	8	(3.9)
	P	P	NT	P	12	(5.8)
	P	P	P	NT	1	(0.5)
	P	P	N	N	3	(1.5)
	P	P	N	NT	3	(1.5)
	P	P	NT	NT	3	(1.5)
	P	N	N	P	2	(1.0)
	P	P	N	P	44	(21.4)
	P	GZ	N	P	1	(0.5)
	P	N	NT	P	2	(1.0)
	P	GZ	NT	P	1	(0.5)
	P	GZ	NT	NT	1	(0.5)
	N	P	N	N	11	(5.3)
	N	P	N	ND	1	(0.5)
	N	P	NT	N	2	(1.0)
	N	P	P	N	2	(1.0)
	N	P	P	P	3	(1.5)
	N	P	N	P	41	(19.9)
	N	P	NT	P	21	(10.2)
	N	P	NT	NT	6	(2.9)
	N	N	P	P	2	(1.0)
	N	GZ	P	P	1	(0.5)
	N	N	N	P	18	(8.7)
	N	GZ	N	P	1	(0.5)
	N	N	NT	P	6	(2.9)
*Negative:*					*4*	*(1.9)*
	N	N	N	N	4	(1.9)
*Undefined:*					*3*	*(1.5)*
	N	N	NT	N	1	(0.5)
	N	N	NT	NT	2	(1.0)
TOTAL (%)					206	(100)
Positive^4^	81/206(39)	161/206(78)	17/146(12)	166/189(88)	199/203	(98)

1 Culture of spleen aspirate;

2 leishmanin skin test;

3 PCR of spleen aspirate;

4 The denominator varies according with the number of animals subjected to the test. N = negative test; P = positive test; GZ = dubious test result; NT = not tested.

### Histological Disorganization of splenic white Pulp and Clinical Signs of Disease in Dogs with Evidence of Leishmania Infection

Of the 199 dogs with evidence of *Leishmania* infection, 54 (27%) had spleen type 1, 99 (50%) had spleen type 2, and 46 (23%) had spleen type 3 (Table 3). Among the 4 animals without evidence of *Leishmania* infection, 2 (50%) had spleen type 1, and 2 (50%) had spleen type 2.

**Table 3:** Distribution of clinical signs associated with visceral leishmaniasis in dogs with evidence of infection by *L. infantum* and type 1 (normal) or type 3 (moderately to severely disorganized) splenic white pulp.

PARAMETER	SPLEEN TYPE 1		SPLEEN TYPE 2		SPLEEN TYPE 3		TOTAL (%)	
*N*	54	(100)	99	(100)	46	(100)	199	(100)
Spleen type:								
Type 1	54	(100)					54	(27)
Type 2			99	(100)			99	(50)
Type 3					46	(100)	46	(23)
Clinical classification:								
Asymptomatic	10/54	(18)	11/99	(11)	1/46	(2)	22/199	(11)
Symptomatic	44/54	(82)	88/99	(89)	45/46	(98)^b^	177/199	(89)
Clinical signs:								
Alopecia	14/54	(26)	44/99	(44)	25/46	(54)^c^	83/199	(42)
Anemia	10/33	(30)	31/70	(44)	22/36	(61)^a^	63/139	(45)
Conjunctivitis	9/28	(32)	15/47	(32)	17/30	(57)	41/105	(39)
Dehydration	5/28	(18)	19/56	(34)	11/23	(48)^a^	35/107	(33)
Dermatitis	6/26	(23)	24/43	(56)	13/23	(56)^a^	43/92	(47)
Emaciation	19/54	(35)	43/99	(43)	24/46	(52)	86/199	(43)
Erosion	8/28	(29)	18/56	(32)	9/23	(39)	35/107	(33)
Lymphadenopathy	23/52	(44)	58/99	(59)	34/46	(74)^c^	115/197	(58)
Onychogryphosis	7/54	(13)	29/97	(30)	16/45	(36)^b^	52/196	(27)
Pustule	3/28	(11)	12/56	(21)	2/23	(9)	17/107	(16)
Splenomegaly	20/33	(61)	40/66	(61)	15/26	(58)	75/125	(60)
Ulceration	4/28	(14)	8/56	(14)	7/23	(30)	19/107	(18)

Chi-square test for trends:

a P<0.05,

b P<0.01,

c P<0.005. The denominator varies according to the availability of the recorded data for each clinical sign.

The proportion of symptomatic animals was higher among the dogs with evidence of *Leishmania* infection and spleen type 3 (45/46, 98%) than among the dogs with evidence of *Leishmania* infection and spleen type 1 (44/54, 82%, Chi-square test for trend, P<0.01). Among the 4 animals without evidence of *Leishmania* infection, 2 were asymptomatic, and 2 were symptomatic.

**Figure 1:**
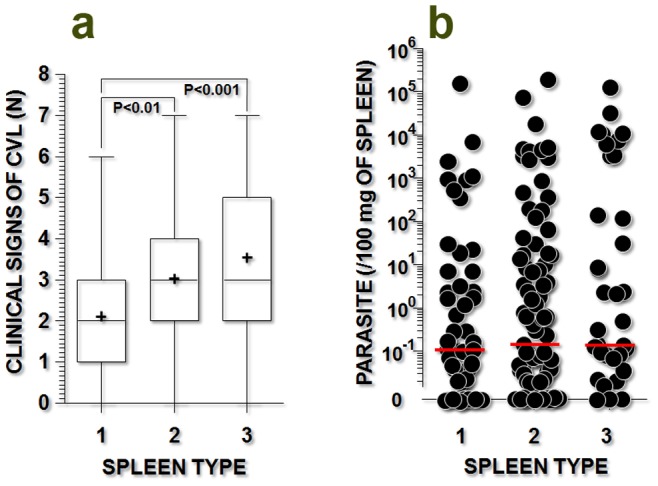
Clinical signs of visceral leishmaniasis and parasite burden in dogs naturally infected with *Leishmania infantum* and with different levels of disorganization of the splenic white pulp. **a)** frequency of clinical signs associated with visceral leishmaniasis (alopecia, anemia, conjunctivitis, dermatitis, emaciation, lymphadenopathy, and onychogryphosis); **b)** parasite burden in the spleen, as estimated by qPCR (see details in the text). The spleen types include spleen type 1 (organized), type 2 (slightly disorganized), and type 3 (moderately to severely disorganized). The Kruskal-Wallis statistical test was used.

The number of clinical signs associated with visceral leishmaniasis (median, interquartile range) was higher in animals with evidence of *Leishmania* infection and spleen type 3 (3, 2–5, P<0.001) or type 2 (3, 2–4, P<0.01) than in animals with spleen type 1 (2, 1–3, Kruskal-Wallis test, Fig. 1a). Alopecia, anemia, dehydration, dermatitis, lymphadenopathy, and onychogryphosis were all more frequent among animals with evidence of *Leishmania* infection and spleen type 3 than among those with spleen type 1 (Chi-square test, P<0.05, Table 3). However, the parasite burden was similar among the animal groups irrespective of the level of organization of their splenic lymphoid tissue (Fig. 1b).

### Clinical Signs of Visceral Leishmaniasis in Dogs with Active *L. Infantum* Infection and white Pulp Disorganization

Although most of the animals had evidence of *Leishmania* infection, some of the diagnostic tests used in this study, such as PCR and LST, may not reflect the susceptibility of these animals to visceral leishmaniasis. The LST may even reflect some degree of protection against the disease [12]. Conversely, positive serology and a positive spleen culture are both indicative of an active infection [21], [24]. Therefore, we investigated the association between white pulp disorganization and the clinical signs of visceral leishmaniasis in dogs with or without a positive spleen culture.

**Figure 2:**
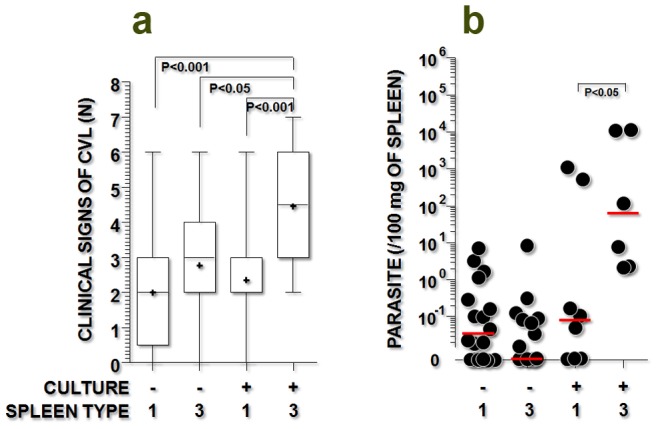
Clinical signs of visceral leishmaniasis and parasite burden in dogs with active (positive spleen culture) or quiescent (negative spleen culture) infection by *Leishmania infantum*, with different levels of disorganization of the splenic white pulp. **a)** frequency of clinical signs associated with visceral leishmaniasis (alopecia, anemia, conjunctivitis, dermatitis, emaciation, lymphadenopathy, and onychogryphosis); **b)** parasite burden in the spleen, as estimated by qPCR (see details in the text). The spleen types include spleen type 1 (organized) and type 3 (moderately to severely disorganized). The Kruskal-Wallis statistical test was used.

The number of clinical signs associated with visceral leishmaniasis (median, interquartile range) was higher in animals with a positive spleen culture and a type 3 spleen (4.5, 3–6) than in animals with a negative spleen culture and a type 1 spleen (2, 0.5–3, P<0.001), animals with a negative spleen culture and a type 3 spleen (3, 2–4, P<0.05), or animals with a positive spleen culture and a type 1 spleen (2, 2–3, P<0.001, Kruskal-Wallis test, Fig. 2a).

As shown in Table 4, the chance of developing alopecia (OR = 12.2, CI = 3.3–44.8), anemia (OR = 4.4, CI = 1,2–16.3), conjunctivitis (OR = 5.3, CI = 1.2–23.3), dehydration (OR = 21.0, CI = 2.9–153.8), dermatitis (OR = 6.9, CI = 1.3–37.2), emaciation (OR = 9.7, CI = 2.8–32.8), erosion (OR = 6.4, CI = 1.2–35.4), onychogryphosis (OR = 6.2, CI = 1.8–20.8), and ulceration (OR = 16.0, CI = 1.4–176.6) was higher in animals with a positive spleen culture and a type 3 spleen than in animals with a negative spleen culture and normal white pulp. Animals with a negative spleen culture and a type 3 spleen or with a positive spleen culture and a type 1 spleen also had a less severe disease presentation than animals with a positive spleen culture and a type 3 spleen (Table 4). Furthermore, the associated risk of presenting any of the clinical signs of visceral leishmaniasis was higher in the animals with an active infection and a spleen type 3 than in the other groups (Table 4).

**Table 4:** Distribution of clinical signs associated with visceral leishmaniasis in dogs with quiescent (negative spleen culture) or active (positive spleen culture) infection by *L. infantum* and type 1 (normal) or type 3 (moderately to severely disorganized) splenic white pulp (WP).

	NEGATIVE SPLEEN CULTURE						POSITIVE SPLEEN CULTURE							
CLINICAL SIGNS	WP TYPE 1		WP TYPE 3				WP TYPE 1				WP TYPE 3			
	N	(%)	N	(%)	*OR*	[95% CI]	N	(%)	*OR*	[95% CI]	N	(%)	*OR*	[95% CI]
Alopecia	10/37	(27)	8/26	(31)	*1.2*	[0.4–3.6]	6/20	(30)	*1.2*	[0.4–3.8]	18/22	(82)	*12.2*	[3.3–44.8]^d^
Anemia	9/23	(39)	10/19	(53)	*1.7*	[0.5–5.9]	2/12	(17)	*0.3*	[0.0–1.7]	14/19	(74)	*4.4*	[1.2–16.3]^a^
Conjunctivitis	5/17	(29)	7/15	(46)	*2.1*	[0.5–9.0]	4/12	(33)	*1.2*	[0.2–5.9]	11/16	(69)	*5.3*	[1.2–23.3]^b^
Dehydration	3/21	(14)	4/16	(25)	*2.0*	[0.4–10.6]	3/9	(33)	*3.0*	[0.5–19.0]	7/9	(77)	*21.0*	[2.9–153.8]^c^
Dermatitis	3/16	(19)	5/10	(50)	*4.3*	[0.7–25.3]	4/11	(36)	*2.5*	[0.4–14.4]	8/13	(62)	*6.9*	[1.3–37.2]^a^
Emaciation	8/37	(22)	8/26	(31)	*1.6*	[0.5–5.0]	12/20	(60)	*5.4*	[1.6–17.9]^b^	16/22	(73)	*9.7*	[2.8–32.8]^c^
Erosion	5/21	(24)	4/16	(25)	*1.1*	[0.2–4.8]	4/9	(44)	*2.6*	[0.5–13.4]	6/9	(67)	*6.4*	[1.2–35.4]^a^
Lymphadenopathy	18/35	(51)	20/26	(77)	*3.2*	[1.0–9.7]	7/20	(35)	*0.5*	[0.2–1.6]	16/22	(72)	*2.5*	[0.8–8.0]
Onychogryphosis	6/37	(16)	4/25	(16)	*1.0*	[0.2–3.9]	2/20	(10)	*0.6*	[0.1–3.2]	12/22	(54)	*6.2*	[1.8–20.8]^b^
Pustule	2/21	(10)	1/16	(6)	*0.6*	[0.0–7.7]	1/9	(11)	*1.2*	[0.1–15.0]	1/9	(11)	*1.2*	[0.1–15.0]
Splenomegaly	13/24	(54)	10/17	(59)	*1.2*	[0.3–4.2]	8/11	(73)	*2.3*	[0.5–10.6]	7/11	(64)	*1.5*	[0.3–6.4]
Ulceration	1/21	(5)	3/16	(19)	*4.6*	[0.4–49.3]	3/9	(33)	*10.0*	[0.9–114.8]	4/9	(44)	*16.0*	[1.4–176.6]^a^

OR = odds ratio; CI = confidence interval;

a P<0.05;

b P<0.01;

c P<0.005;

d P<0.001 (Chi-square test or Fisher’s exact probability test).

The splenic parasite burden (median, interquartile range) in animals with a positive spleen culture and a type 3 spleen (62.2, 2.3–11168.0) was higher than that observed in animals with a positive spleen culture and a type 1 spleen (0.1, 0.0–395.6, P<0.05, Mann-Whitney test, Fig. 2b).

### Laboratory Tests in Dogs with Active *L. Infantum* Infection and white Pulp Disorganization

The serum levels of albumin (median, interquartile range) were lower in animals with a positive spleen culture and a type 3 spleen (1.8, 1.4–2.3 g/dL) than in animals from the other groups (Table 5). The serum levels of creatinine were lower in animals with a positive spleen culture and a type 3 spleen (0.7, 0.4–0.8 mg/dL) than in animals with a negative spleen culture and a type 1 spleen (0.9, 0.7–1.2 mg/dL, Kruskal-Wallis test, P<0.05, Table 5). The serum creatinine levels were also lower in animals with a positive spleen culture and a type 3 spleen (0.7, 0.4–0.8 mg/dL) than in animals with a type 1 spleen and either a negative (0.9, 0.7–1.2 mg/dL, Kruskal-Wallis test, P<0.05) or positive (1.0, 0.8–1.1 mg/dL, Kruskal-Wallis test, P<0.01) spleen culture. The serum levels of ALT were higher in dogs with evidence of *Leishmania* infection and a type 3 spleen (53±32 U) than in infected animals with spleen type 1 (27±9 U, Kruskal-Wallis test, P<0.05, Table 5). There were no significant differences between the groups for the other biochemical or hematological tests.

**Table 5:** Distribution of laboratory results in dogs with quiescent (negative spleen culture) or active (positive spleen culture) infection by *L. infantum* and type 1 (normal) or type 3 (moderately to severely disorganized) splenic white pulp (WP).

CLINICAL	WITH NEGATIVE SPLEEN CULTURE				WITH POSITIVE SPLEEN CULTURE			
PARAMETER	SPLEEN TYPE 1		SPLEEN TYPE 3		SPLEEN TYPE 1		SPLEEN TYPE 3	
	MEDIAN	[p25–p75; *N*]	MEDIAN	[p25–p75; *N*]	MEDIAN	[p25–p75; *N*]	MEDIAN	[p25–p75; *N*]
Albumin	2.8	[2.4–3.4; *21*]^c^	2.6	[2.5–3.2; *16*]^b^	2.7	[2.2–4.0; *9*]^b’^	1.8	[1.4–2.3; *8*]^b,b’,^ c
Globulin	4.8	[3.9–7.2; *21*]	7.0	[5.2–9.7; *16*]	4.6	[4.0–6.6; *9*]	5.7	[4.0–6.5; *8*]
ALT	23	[20]–[32]; [*9*] a	36	[29–46; *11*]	26	[25]–[27]; [*2*]	57	[38–95; *5*]^a^
AST	34	[30–41; *9*]	37	[25–50; *11*]	32	[27]–[37]; [*2*]	40	[34–63; *5*]
Urea	30	[22–42; *21*]	30	[25–44; *16*]	46	[30–54; *9*]	41	[22–45; *8*]
Creatinine	0.9	[0.7–1.2; *21*]^a^	0.8	[0.7–0.9; *16*]	1.0	[0.8–1.1; *9*]^b^	0.7	[0.4–0.8; *8*]a,b
Cholesterol	162	[142–183; *9*]	180	[161–203; *11*]	180	[142–218; *2*]	238	[136–358; *5*]
Triglycerides	42	[31–74; *9*]	55	[43–98; *11*]	76	[68–85; *2*]	41	[40–72; *5*]
C-reactive protein	28	[9–54; *9*]	ND		32	[17–40; *10*]	44	[39–48; *10*]
Hematocrit	38	[27–43; *9*]^a^	31	[29]–[38]; [*11*]	42		19	[17]–[27]; [*5*] a
Hemoglobin	13	[9]–[14;*9*] a	11	[10]–[12;*11*]	14		7	[6]–[9]; [*5*] a
WBC count	14	[13]–[15]; [*9*]	14	[8]–[19;*11*]	18		28	[15–46; *5*]
Neutrophils	60	[42–74; *9*]^a^	68	[49–78; *11*]	36		82	[80–83; *4*]^a^
Monocytes	5	[3]–[7]; [*14*]	2	[2]–[3]; [4]	8	[8]–[9]; [*2*]	4	[1]–[6;6;*4*]
Lymphocytes	16	[12]–[24;*14*]	43	[14–77; *4*]	22	[12]–[31]; [*2*]	8	[4]–[18;*6*]
Eosinophils	4	[1]–[8]; [*14*]	1	[0–4; *4*]	12	[10]–[14]; [*2*]	2	[1]–[9;*6*]
Platelets	130	[57–168; *9*]	161	[96–232; *11*]	161		155	[80–375; *5*]

p25–p75 = interquartile range; ALT = alanine aminotransferase; AST = aspartate aminotransferase;

a P<0.05,

b, b’ P<0.01,

c P<0.001 (Kruskal-Wallis test): animal groups with significant differences in the laboratory test are identified by the same character (^a^, ^b^,^ b’^, or^ c^).

## Discussion

The prevalence of *L. infantum* infection in dogs is endemically high in some areas of Brazil. In a recent survey of the prevalence of infection in stray dogs collected from the streets of Jequié, Bahia State, Brazil, as estimated by culture, serology, and LST (DNA techniques were not used), up to 87% of animals tested positive for infection [25]. For the sample used in this study, when qPCR was used in addition to those tests, the estimated prevalence reached 98%. A similar prevalence has been reported by Berrehal and colleagues (1996) in asymptomatic dogs from an endemic area of Marseille, France [26]. Hence, this work supports the development of future studies with an appropriate design to estimate the actual prevalence of infection in the street dogs of endemic areas in Brazil.

Moderate to extensive disorganization of the splenic white pulp was observed in nearly 25% of the animals, all of which had evidence of *Leishmania* infection. However, the number of uninfected animals was low (only 4 of 203 dogs were negative in all performed tests), preventing comparison between the infected and uninfected groups.

Nevertheless, herein we demonstrate that in the animals with evidence of *Leishmania* infection, the disorganization of the splenic lymphoid tissue is associated with the clinical and laboratory features of more severe disease, supporting the hypothesis of this study.

Animals with evidence of *Leishmania* infection and spleen type 3 had more clinical symptoms attributed to visceral leishmaniasis, including alopecia, anemia, conjunctivitis, dehydration, dermatitis, emaciation, erosion, and onychogryphosis, than infected animals with normal spleens. The association between the severity of canine visceral leishmaniasis and the disorganization of the splenic lymphoid tissue was even more evident when the group of animals with evidence of active infection (confirmed by a positive spleen culture) was considered. In these animals, the disorganization of the splenic lymphoid tissue was associated with a higher frequency of the clinical features mentioned above. These animals also had a higher frequency of lymphadenopathy and ulceration than the animals in the other groups. Furthermore, these animals had lower levels of serum albumin and creatinine and slight increases in ALT levels compared with the animals from the other groups.

The typical pattern of lymphoid tissue changes reported in dogs, humans, and mice infected with *Leishmania* is characteristic of lymphoid tissue hyperplasia [27], [28], [29]. This hyperplasia may result from the reaction of B and T cells to the *Leishmania* antigen and is associated with lymphocyte proliferation in the B- and T-cell areas of the lymphoid organs and with macrophage infiltration [27], [28], [29]. In the spleen, these hyperplastic changes are more prominent in the B-cell compartments [28], [29]. However, observations in humans who have died of visceral leishmaniasis, mice with severe infection, and dogs with severe disease have shown that atrophy and the disruption of splenic histology also occur during the course of the disease [12], [13], [17], [30], [31], [32]. Studies by Veres and colleagues (1983) and by Cobertt and colleagues using murine models of visceral leishmaniasis clearly demonstrated that the secondary lymphoid tissues undergo sequential changes in the course of visceral leishmaniasis, passing from hyperplasia to atrophy and lymphoid depletion [9], [28]. We previously demonstrated that 45% of naturally infected dogs have normal or hyperplastic splenic lymphoid tissue whereas 55% have some degree of splenic lymphoid tissue disorganization and atrophy [12].

The factors that determine this disorganization of lymphoid tissue in visceral leishmaniasis are not completely understood. Evidence suggests that the *Leishmania* infection itself may be responsible for this alteration [10], [33]. The loss of specific cell populations, such as marginal zone macrophages, T lymphocytes, and follicular dendritic cells, may be the original cause of the lymphoid tissue disorganization, as shown in experimental models of visceral leishmaniasis [10], [33]. Marginal zone macrophages and follicular dendritic cells are involved in signaling pathways that control B-lymphocyte migration into the lymphoid follicle [34]. The follicular dendritic cell–CXCL13–B lymphocyte pathway of lymphoid follicle assembly is altered in the spleens of dogs with visceral leishmaniasis and lymphoid tissue disorganization [13]. Notably, in a study of mice infected with *L. infantum,* Carrion et al. observed an association between the size of the inoculum and lymphoid tissue disruption [32]. A similar association was also observed in our study among animals with a positive spleen culture, although a considerable overlap in parasite burden was observed between infected animals with type 1 or type 3 spleens. This finding may indicate that other factors, such as co-infections, also contribute to the splenic disruption in stray dogs.

Given the characteristics of this cross-sectional study, defining a causal relationship between the observed splenic changes and the aggravation of visceral leishmaniasis in these animals is not possible. Some of the conditions, such as weight loss, that were observed in the dogs with severe canine visceral leishmaniasis in this study may lead to disorganization of the lymphoid tissue in the spleen [35]. However, only 52% of the animals with a substantial disruption of the splenic lymphoid tissue (type 3 spleen) were emaciated. Other infections may also lead to a disruption of the lymphoid tissue microenvironments [8]. Canine distemper, an endemic viral disease in Brazil, is also associated with lymphoid tissue disruption [11]. A complete survey of other canine infections was not performed in this study, but these animals live in packs, making them more likely to acquire a variety of infections that are endemic among stray dogs in Brazil. Therefore, infections by other pathogens may contribute to the splenic lymphoid tissue disorganization observed in these animals. For example, distemper virus infection has been shown to cause lymphoid tissue disruption and immunodeficiency in dogs [36], [37]. The neutrophil counts and ALT levels were higher in animals with active *Leishmania* infection and type 3 spleens. These animals also exhibited a trend toward an increase in C-reactive protein levels. Such evidence of an inflammatory state in these animals may indicate acute infection by other pathogens, such as purulent conjunctivitis. Further work is needed to examine the contribution of these co-infections to the development of visceral leishmaniasis in *Leishmania*-infected dogs and the role played by lymphoid organ disorganization in this mechanism of disease.

Previous work by our group and others has shown that such disorganization of the splenic lymphoid tissue is associated with other laboratory markers of severe visceral leishmaniasis in dogs, including positive spleen culture, higher levels of serum antibodies against *Leishmania*, and negative LST results [12]. The lymphoid follicle and marginal zone are the splenic lymphoid compartments that are most severely affected by the disorganization associated with canine visceral leishmaniasis [13], [17]. These 2 spleen compartments play an important role in host defense against bacterial infection. The splenic marginal zone is also the site of the homing of a variety of memory B cell populations involved in the response against T-dependent and T-independent antigens [1]. Thus, the disorganization of the microenvironments of these splenic compartments may interfere with the immune response to bacterial infections, such as purulent conjunctivitis, and with the inflammatory state observed in these animals. Humans who die of severe visceral leishmaniasis also present with concomitant spleen lymphoid tissue disruption and bacterial infections in the lung, ear, oral mucosa, intestinal tract, and skin [30], [38]. Further studies are needed to examine the potential changes in the immune response to infections by other pathogens, such as bacteria and fungi, in animals with disrupted lymphoid tissue.
